# β-Catenin signaling in hepatocellular carcinoma

**DOI:** 10.1172/JCI154515

**Published:** 2022-02-15

**Authors:** Chuanrui Xu, Zhong Xu, Yi Zhang, Matthias Evert, Diego F. Calvisi, Xin Chen

**Affiliations:** 1 School of Pharmacy, Tongji Medical College, Huazhong University of Science and Technology, Wuhan, China.; 2 Department of Gastroenterology, Zhongnan Hospital of Wuhan University, Wuhan, China.; 3 Key Laboratory of Biorheological Science and Technology, Ministry of Education, College of Bioengineering, Chongqing University, Chongqing, China.; 4 Institute of Pathology, University of Regensburg, Regensburg, Germany.; 5 Department of Bioengineering and Therapeutic Sciences and Liver Center, UCSF, San Francisco, California, USA.

## Abstract

Deregulated Wnt/β-catenin signaling is one of the main genetic alterations in human hepatocellular carcinoma (HCC). Comprehensive genomic analyses have revealed that gain-of-function mutation of *CTNNB1*, which encodes β-catenin, and loss-of-function mutation of *AXIN1* occur in approximately 35% of human HCC samples. Human HCCs with activation of the Wnt/β-catenin pathway demonstrate unique gene expression patterns and pathological features. Activated Wnt/β-catenin synergizes with multiple signaling cascades to drive HCC formation, and it functions through its downstream effectors. Therefore, strategies targeting Wnt/β-catenin have been pursued as possible therapeutics against HCC. Here, we review the genetic alterations and oncogenic roles of aberrant Wnt/β-catenin signaling during hepatocarcinogenesis. In addition, we discuss the implication of this pathway in HCC diagnosis, classification, and personalized treatment.

## Introduction

Hepatocellular carcinoma (HCC) is the most common form of liver cancer, representing the third leading cause of cancer-related death (1). The incidence of HCC ranks sixth among all tumor types worldwide. Increased HCC occurrence in this decade reflects persistent hepatitis B and C virus infection and the increase of nonalcoholic steatohepatitis (NASH) since 2000 (2). A projection study indicated that the age-standardized incidence rates per 100,000 person-years for primary liver cancer would increase in both men and women by the year 2030 in most countries as a result of increased NAFLD and/or NASH (3). During the past decade, multitargeted tyrosine kinase inhibitors (TKIs), such as sorafenib, lenvatinib, regorafenib, and cabozantinib, have been used as first- or second-line drugs for patients with unresectable HCC (4). However, these agents provide limited survival benefits and are associated with considerable toxicities and poor quality-of-life outcomes. Immune checkpoint inhibitors (ICIs) have been approved for HCC treatment and show a similar response rate (15%–30%) compared with TKI therapies (5). For example, HCC patients who received the CTLA4-blocking ICI tremelimumab showed a partial response rate of 18% and a disease control rate of 76% (6). PD-1 and PD-L1 blockade showed higher objective response rates, which could reach 20% in advanced HCC patients (7). Recently, the phase III IMbrave150 trial results showed that combining an anti–PD-L1 antibody with an anti–VEGF-A antibody leads to promising efficacy for advanced HCC patients (8). Currently, this combination immunotherapy has become the first-line treatment strategy against HCC (9). Nevertheless, most patients eventually progress under this regimen. Therefore, studies to elucidate the molecular mechanisms underlying HCC pathogenesis are imperative to develop additional and more effective drugs for precision medicine.

## Molecular mechanisms of Wnt/β-catenin activation in HCC

**Figure 1:**
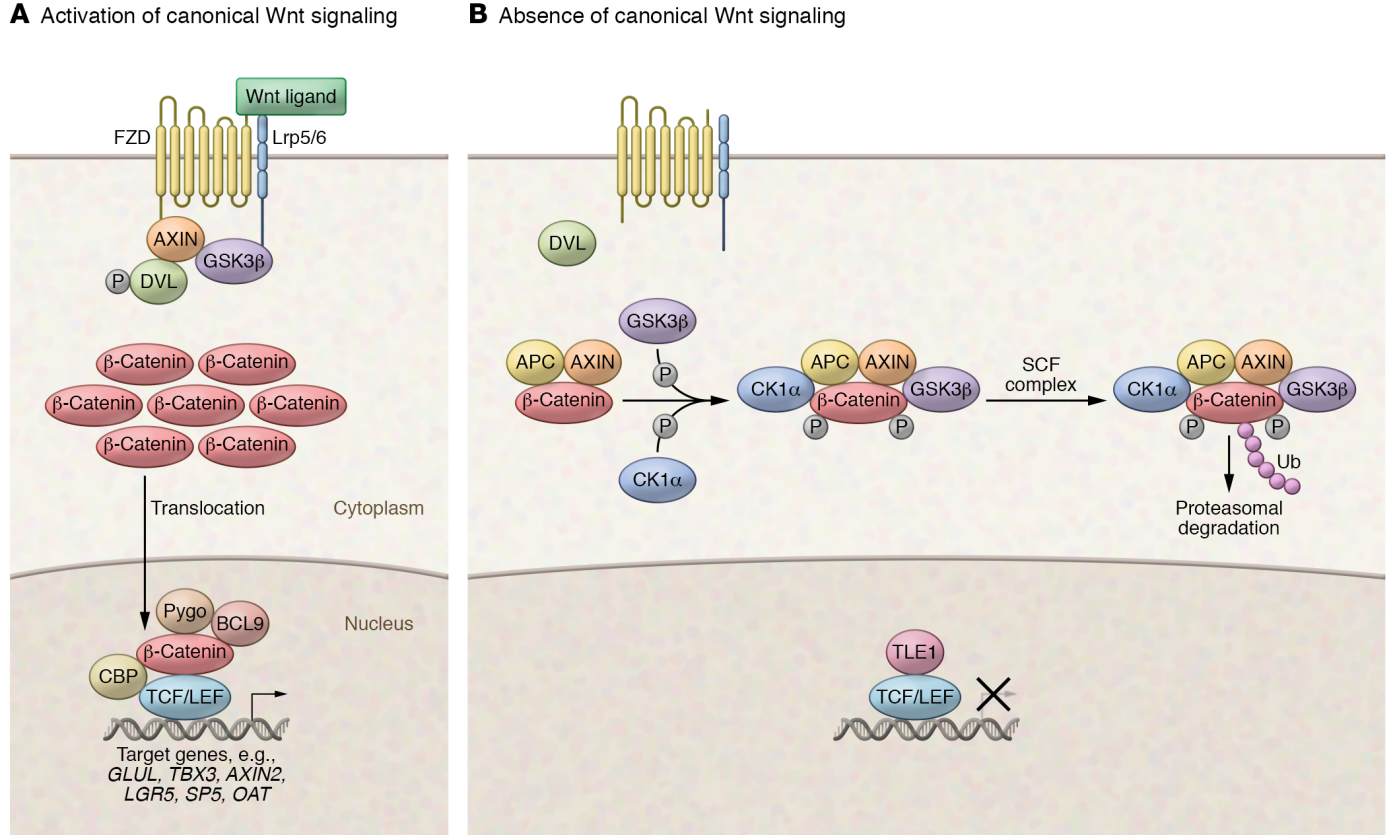
Canonical Wnt/β-catenin signaling pathway in HCC. (**A**) When Wnt ligands are present, Wnt/FZD signaling activation leads to the phosphorylation of mammalian homolog of dishevelled (DVL). Phosphorylated DVL recruits AXIN and GSK3β to the plasma membrane, hence blocking the degradation complex’s formation. Subsequently, β-catenin accumulates in the cytoplasm and then translocates into the nucleus. Nuclear β-catenin binds to TCF/LEF transcription factors and promotes the transcription of target genes. (**B**) When Wnt ligands are absent, soluble β-catenin is phosphorylated by the GSK3β-CK1α-APC-AXIN1 complex. Once phosphorylated, β-catenin is degraded by the proteasome after ubiquitination by the Skp-, Cullin-, and F-box–containing (SCF) protein complex. When β-catenin is absent in the nucleus, the TCF/LEF transcription factors are repressed by TLE-1. *CTNNB1* (encoding β-catenin), *AXIN1*, and *APC* are mutated in 27%, 8%, and 3% of human HCCs, respectively.

The Wnt/β-catenin cascade is one of the major signaling pathways regulating liver homeostasis, regeneration, and tumorigenesis (10), which has been extensively reviewed (11, 12). In brief, in the absence of Wnt ligands, most cellular β-catenin is sequestered in the adherens junctions at the plasma membrane (Figure 1). Cytosolic β-catenin associates in a complex with adenomatous polyposis coli (APC) and AXIN1 proteins, which mediate the N-terminal phosphorylation of β-catenin. This event leads to the ubiquitination of β-catenin by the E3 ubiquitin ligase β-transducin repeat–containing protein (β-TRCP) and subsequent proteasomal degradation. When Wnt ligands bind to the Frizzled receptors, Dvl/Dsh is phosphorylated and, in turn, recruits AXIN1 and GSK3β adjacent to the plasma membrane, thus preventing the formation of the degradation complex. As a result, unphosphorylated β-catenin escapes recognition by β-TRCP and translocates into the nucleus, where it binds to the T cell factor (TCF) and lymphoid enhancer–binding protein family (LEF) transcription factors. The activated β-catenin/TCF/LEF complex induces the transcription of genes regulating cell proliferation and survival (Figure 1).

In the normal liver, β-catenin is membrane-localized in hepatocytes, and the Wnt/β-catenin pathway is activated in pericentral hepatocytes, which is demonstrated by β-catenin–dependent glutamine synthetase (GS) staining in these cells (13, 14). In HCC, recent genomic studies revealed that 30% to 40% of tumors demonstrate aberrant activation of the Wnt/β-catenin cascade (15). The activation of this pathway could be subdivided into somatic genetic events and nongenetic events. For somatic mutations leading to Wnt/β-catenin activations, The Cancer Genome Atlas (TCGA) analysis reveals that gain-of-function (GOF) mutations of *CTNNB1*, which encodes β-catenin, occur in 27% of HCC patients (Figure 1). Most *CTNNB1* missense mutations arise at the serine/threonine sites of exon 3 or adjacent amino acids, which prevents the β-catenin protein from phosphorylation and degradation, leading to its stabilization and unrestrained transcriptional activity (14). In addition, mutations in armadillo repeat domains 5 and 6 of the β-catenin protein are also frequently observed in human HCCs (16). Studies have suggested that these amino acid substitutions have reduced binding to APC, leading to increased Wnt/β-catenin signaling (16). Mutations have also been observed in *APC* and *AXIN1*, encoding critical components of the β-catenin degradation complex. Mutations in *APC* and *AXIN1* are found in 3% and 8% of HCC, respectively (Figure 1). *APC* and *AXIN1* mutations are mostly missense, deleted, and/or truncated mutations, resulting in loss of protein expression and function, a characteristic of tumor suppressors (17). Importantly, mutations of *CTNNB1*, *APC*, or *AXIN1* rarely co-occur in the same HCC, suggesting that these mutations lead to common downstream effectors. Notably, HCC patients harboring GOF *CTNNB1* mutations demonstrate robust upregulation of canonical Wnt target genes, including *GLUL*, *TBX3*, *AXIN2*, *LGR5*, *SP5*, and *OAT* (Figure 1).

Studies have also revealed multiple nongenetic mechanisms leading to Wnt/β-catenin activation. These include promoter hypermethylation and related silencing of the secreted Frizzled-related protein 1 gene (*SFRP1*), a Wnt/β-catenin antagonist (18); overexpression of Frizzled (FZD) membrane receptor and Wnt ligands (19); and deregulated expression of microRNAs (20) and long noncoding RNAs (21) that regulate Wnt/β-catenin signaling.

## Unique features of HCC with Wnt/β-catenin activation

Studies have illustrated that human HCCs with aberrant Wnt/β-catenin activation have distinct clinical, pathological, and molecular features. Multiple investigations suggest that overexpression and mutations of β-catenin occur more frequently in HCV-related HCCs than in HBV-related HCCs (22–24) and are commonly observed in HCC with noncirrhotic liver in the absence of usual HCC risk factors (25, 26). Activation of the Wnt/β-catenin cascade has been linked to early-stage HCC (24, 27), but also tumor progression (28). Association between β-catenin activation and HCC patient survival remains controversial, with most studies suggesting that *CTNNB1* mutation is a favorable prognostic marker. For instance, using meta-analysis, Wang et al. reported that HCC patients with *CTNNB1* mutations demonstrate a more prolonged overall survival (29). Similar results came from a study by Ding et al. (30). However, Lu and colleagues reported that *CTNNB1* mutations are not associated with prognosis in advanced HCC (31).

The histopathological features of human HCC lesions with β-catenin activation have also been extensively investigated, providing conflicting results. For instance, Hsu et al. showed that β-catenin mutations are associated with grade I HCC (22). In addition, Wong et al. found that HCCs with a non-nuclear type of β-catenin overexpression have poorer cellular differentiation (32). In contrast, there were no significant differences in HCC tumor grade between β-catenin–positive and –negative tumors in two other investigations (33, 34). These discrepancies remain to be addressed and might be due to the different analyses conducted (using either HCCs with β-catenin mutations or nuclear accumulation of the protein for the comparisons) or the lack of a standard and specific delineation of β-catenin–“positive” tumors based on the staining patterns (i.e., the percentage of cells positive for nuclear β-catenin defining an HCC as either β-catenin positive or negative). Finally, Audard et al. were the first to try to outline macroscopic and microscopic features of *CTNNB1*-mutated HCCs (25). They demonstrated that *CTNNB1*-mutated HCCs are usually large (>6 cm in diameter) and solitary lesions. Typical, albeit non-pathognomonic, microscopic features of *CTNNB1*-mutated HCCs are microtrabecular and acinar growth, a high degree of differentiation (Edmondson grade G1–G2), homogeneous microscopic appearance, prominent cholestasis, and lack of steatosis and inflammation. Interestingly, they showed that robust and uniform immunohistochemical expression of glutamine synthetase (GS), a target of the Wnt/β-catenin pathway, was more sensitive (90%) than cytoplasmic/nuclear β-catenin positivity (63%) in identifying *CTNNB1*-mutated HCCs, though with equal specificity (both 98%). Indeed, based on TCGA analysis, the upregulation of *GLUL*, which encodes GS, and other canonical Wnt/β-catenin target genes is strongly associated with *CTNNB1* mutation status in HCC (Figure 1). These results were confirmed by Calderaro et al. in a large study comparing the correlation of morphology and molecular features in a large cohort of HCCs (35).

Overall, human HCCs can be subdivided into two major groups: a proliferation group and a nonproliferation group (36, 37). Each of these groups accounts for approximately 50% of human HCCs and consists of several subgroups identified in various genomic studies (Figure 1B). In addition, based on TCGA studies, HCC could be classified into clusters 1, 2, and 3 (38). Clusters 1 and 3 belong to the proliferation group and cluster 2 to the nonproliferation group. Boyault et al. further defined human HCCs into G1 to G6 subgroups (39). Among them, G1, G2, and G3 are classified as proliferation group, whereas G4, G5, and G6 are defined as nonproliferation group. The proliferation group and the nonproliferation group show different molecular, genetic, epigenetic, and clinical features. The proliferation group is associated with chromosomal instability, DNA hypomethylation, alcohol- or HCV-related HCC, low serum α-fetoprotein levels, and low frequency of vascular invasion. In contrast, the nonproliferation group is characterized by chromosomal stability, promoter hypermethylation, frequent HBV infection, more aggressive phenotype, poor tumor differentiation, high serum α-fetoprotein levels, and increased vascular invasion (40). Intriguingly, GOF *CTNNB1* mutations are frequently found in the nonproliferation group, and are associated with cluster 2 and G5/G6 subgroups (Figure 1B). In contrast, HCCs with *AXIN1* mutations belong to the proliferation group, and are associated with cluster 1 and G1 subgroups (Figure 1B).

## Induction of hepatocarcinogenesis by Wnt/β-catenin

Activated Wnt/β-catenin signaling has been considered an early signaling event in HCC pathogenesis (41, 42). Importantly, studies have shown that *CTNNB1* mutation is one of the significant key genetic events in human HCCs (43, 44). Furthermore, Wnt/β-catenin has also been implicated in HCC stemness, progression, metastasis, and drug resistance (45–49). For instance, this pathway has been identified as the prominent signaling that causes the proliferation of cancer stem cells (CSCs). Indeed, overexpression of β-catenin increases self-renewal and in vivo tumorigenicity of HCC CSCs (50–52). Furthermore, activated Wnt/β-catenin has also been associated with resistance to sorafenib and regorafenib in HCC patients (51, 53). All these data support the critical roles of Wnt/β-catenin in various steps of hepatocarcinogenesis.

The oncogenic role of Wnt/β-catenin mutations in HCC was first investigated in transgenic mice. Importantly, transgenic mice overexpressing activated mutant forms of β-catenin develop hepatomegaly, but not HCC (54, 55). These results indicate that activation of Wnt/β-catenin alone may not be sufficient to drive hepatocarcinogenesis. Instead, a second signal is required to cooperate with activated β-catenin to induce HCC development. Consistent with this hypothesis, recent studies using hydrodynamic transfection (56) have demonstrated that oncogenic forms of β-catenin cooperate with other proto-oncogenes such as c-Met (57–59), K-Ras^V12^ (60), activated Akt (61), LKB1 (62), and Nrf2 (63) to induce HCC formation in mice (Table 1). In human HCCs, coordinated activation of c-Met and β-catenin was found in approximately 10% of samples (64). While overexpression of c-Met or the activated mutant form of β-catenin via hydrodynamic injection alone cannot promote HCC formation in mice, coexpression of c-Met and activated β-catenin induces liver tumor development within 6–8 weeks after injection (58). Concomitant *CTNNB1* mutations and *NFE2L2*/*KEAP1* mutations, which lead to action of the Nrf2 pathway, occur in approximately 9% of human HCCs (63). Coexpression of activated forms of β-catenin with mutant NFE2L2, but not the wild-type form of NFE2L2, can induce HCC development in mice (63). Loss-of-function *AXIN1* mutations and c-Met activation were detected in approximately 4% of human HCC, and coexpression of c-Met together with CRISPR/Cas9–based targeting of Axin1 (sgAxin1) in the mouse liver triggers HCC formation (59). Consequent RNA-Seq studies have demonstrated that these murine HCCs share similar gene expression patterns to the subset of human HCCs harboring similar genetic events. In addition, TERT promoter mutations are found in many HCC tissues with *CTNNB1* mutations, indicating a possible synergistic effect of these two genes (65, 66).

Once activated, β-catenin triggers the induction of downstream target expression via the TCF/LEF1 family of transcription factors. Many of these target genes are implicated in hepatocarcinogenesis. c-MYC is one of the best-characterized downstream effectors of β-catenin. However, c-MYC is also regulated by many other mechanisms, such as amplification of the c-MYC locus, increased protein stability, and activation of estrogen receptor, Ras/Raf, and IFN-γ pathways (67–69). *c-MYC* was first identified as a Wnt/β-catenin target gene in the human HT29 colorectal cancer cell line harboring mutant *APC* alleles (70). Subsequently, multiple Wnt response elements were identified in the c-MYC promoter (71). Furthermore, in human HCC, c-MYC could be induced by β-catenin activation (72, 73), and this pathway plays a critical role in gankyrin-driven increased glycolysis and glutaminolysis (74) as well as in sorafenib responsiveness (75).

Cyclin D1 is another direct target of β-catenin and might be a key molecule by which activated β-catenin promotes tumor cell proliferation (76, 77). Numerous studies have demonstrated that activated Wnt/β-catenin induces cyclin D1 expression in mouse and human HCC (78, 79). However, it is worth mentioning that cyclin D1 is not an exclusive effector of the Wnt/β-catenin signaling pathway. Indeed, other molecular cascades could regulate its expression, such as the NF-κB and MAPK pathways (80, 81). Studies conducted in vivo have also illustrated the critical role of cyclin D1 in HCC development (82). Specifically, the coexpression of c-Met and activated mutant forms of β-catenin rapidly induces HCC formation in mice; overexpression of c-Met and cyclin D1 also induces liver tumor development in mice, albeit with longer latency (58). Nevertheless, using *Ccnd1*-knockout mice, Patil et al. showed that cyclin D1 expression is not essential for liver tumor development induced by c-Met and activated mutant forms of β-catenin (58). Mechanistically, cyclin D2 expression in the liver is compensatorily upregulated upon cyclin D1 loss (58). Intriguingly, overexpression of cyclin D1 has also been shown to indirectly enhance the Wnt/β-catenin pathway, leading to increased HCC metastasis (83). Altogether, these studies suggest the interconnected and feedback mechanisms between cyclin D1 and Wnt/β-catenin cascades during hepatocarcinogenesis.

GS, which promotes glutamine synthesis in cells, is a liver-specific Wnt/β-catenin target (84). In normal liver, GS is expressed in a layer of pericentral hepatocytes. Liver-specific knockout of β-catenin in mice leads to complete loss of the pericentral expression of GS (85). As we discussed above, immunostaining of GS may represent a pathological marker for human HCCs with GOF *CTNNB1* mutations (86), although GS expression could also be induced by other factors (87). Studies have shown that GS regulates autophagy downstream of activated β-catenin, which confers sensitivity to sorafenib. Notably, GS-mediated glutamine synthesis is required for *CTNNB1*-mutated HCC growth, since glutamine deprivation inhibits *CTNNB1*-mutated HCC growth in vitro and in vivo (88). Amino acids, including glutamine, are major regulators of mTOR activity in cells (89). Recently, it has been discovered that GS-mediated increased glutamine synthesis leads to mTORC1 activation (90). Accordingly, a strong correlation between activated β-catenin and positive expression of phosphorylated mTOR-S2448 (p–mTOR-S2448) characterizes human HCCs. In addition, *CTNNB1*-mutated HCCs are mTORC1-addicted, owing to the GS/glutamine/p–mTOR-S2448 axis. These studies suggest that mTORC1 inhibitors could be effective for treating *CTNNB1-*mutant and GS-positive human HCCs.

In addition to the genes mentioned above, activated Wnt/β-catenin drives the expression of hundreds of other genes, thus architecting a network of molecules that contributes to tumorigenesis (91, 92). For example, activated Wnt/β-catenin induces the expression of *AXIN2*, which functions as a negative-feedback mechanism to inhibit β-catenin, perhaps avoiding the harmful effects of a completely uncontrolled β-catenin activity (93). *TBX3* is another liver-specific Wnt/β-catenin target gene that can contribute to specific pathological phenotypes via inhibition of the YAP cascade (94). Kinesin family member 2C (KIF2C) is also a direct target of the activated Wnt/β-catenin pathway (95). Its expression is upregulated in HCC and is associated with a poor prognosis. Furthermore, KIF2C enhances mTORC1 activation, providing another link between activated β-catenin and the mTOR cascade in HCC (95). In addition, Wnt/β-catenin is known to induce the expression of multiple matrix metalloproteinases (MMPs), such as MMP2 and MMP9, which contribute to tumor metastasis (96). VEGF-A and VEGF-C, key molecules promoting angiogenesis, are induced by Wnt/β-catenin (97). Moreover, Wnt/β-catenin positively regulates MCL1 expression, associated with sorafenib sensitivity in HCC (98). In addition to activating genes or pathways, Wnt/β-catenin negatively regulates signaling cascades. In the intestine, Wnt inhibits the MAPK pathway (99), whereas, in the liver, it suppresses the NF-κB cascade (100). In mice with liver-specific knockout of *Ctnnb1*, there is increased RelA expression and LPS-induced NF-κB activation (101). However, the inhibitory activities of the Wnt/β-catenin cascade in hepatocarcinogenesis have not been well characterized and require further investigation.

## Targeting Wnt/β-catenin for HCC treatment

Since Wnt pathway activation promotes HCC cell proliferation, migration, and invasion, targeting this signaling cascade is an attractive therapeutic approach for human HCC treatment. Several agents have been screened and investigated for targeting the Wnt pathway in cancer, and some of them are under development. Those agents include small-molecule inhibitors that block the interaction of β-catenin with TCF, such as the fungal derivatives PKF115–854 and CGP049090 (102–106), or the binding of β-catenin to cAMP response element–binding protein (CREB)–binding protein (CBP), such as ICG-001 (107–109). Both PKF115–854 and CGP049090 have shown inhibitory effects against HCC cell growth (45, 106). Therapeutic monoclonal antibodies against Wnts were also developed to block the binding of Wnts to Frizzled (FZ/FZD) receptors, such as anti-Wnt2 monoclonal antibodies (110) and the anti-FZD monoclonal antibody OMP-18R5 (111). Moreover, several approved drugs currently in clinical use have been shown to possess activity against the Wnt pathway (112, 113). These include indomethacin (114, 115), pyrvinium (116), sulindac (117), aspirin (114), celecoxib, and rofecoxib (118). Unfortunately, the antitumor potency of these repurposed drugs has not been established clinically.

In addition to Wnt/TCF inhibitors, agents targeting porcupine (PORCN) or tankyrase (TNKS) have also been developed to block Wnt/β-catenin signaling in cancer cells. PORCN is an *O*-acyltransferase essential for Wnt ligand secretion (119). The PORCN inhibitors, such as LGK-974 (WNT-974) and ETC-159, may inhibit tumor growth via suppression of Wnt signaling. Indeed, studies have shown that LGK-974 can enhance the radiosensitivity of HepG2 cells by modulating Nrf2 signaling (120), and it is investigated in clinical trials for treating various solid tumors (121). TNKS targets AXIN protein for degradation, whereas TNKS inhibition can stabilize AXIN, thus antagonizing Wnt signaling (122). Several TNKS inhibitors with promising therapeutic effects have been developed, including XAV939, G007-LK, G244-LM, RK-287107, JW55, K-756, IWR-1, MSC2504877, AZ1366, JW74, and NVP-TNKS656 (123–132). Preclinical studies have shown that TNKS inhibitors, such as XAV939, can potently inhibit HCC growth in culture (133). However, PORCN and TNKS inhibitors target pathways upstream of β-catenin; therefore, they are unlikely to possess any efficacy against HCCs with GOF *CTNNB1* mutations.

Interfering RNA– or antisense RNA–based therapy is another approach to inhibit the Wnt/β-catenin pathway. In particular, siRNAs targeting Wnts have been shown to suppress HCC cell growth in vitro (134–136). In a GOF *Ctnnb1*-mutant mouse HCC model induced by diethylnitrosamine (DEN) and phenobarbital, use of locked nucleic acid (LNA) antisense oligonucleotides against β-catenin strongly impaired HCC progression (137). In contrast, in the non–*Ctnnb1*-mutant HCC model, induced by DEN only, LNA-si-β-catenin demonstrated no efficacy (137). The therapeutic efficacy of LNA-si-β-catenin has been further validated in vivo in mouse HCCs induced by hydrodynamic transfection of activated forms of K-Ras and β-catenin oncogenes (60).

In summary, various strategies targeting the Wnt/β-catenin cascade have been developed in recent decades. Preclinical studies have provided evidence to support targeting this pathway against cancers, including HCCs. Nevertheless, considerable challenges remain, especially concerning the toxicity of these inhibitors, which suppress the Wnt/β-catenin pathway in normal tissues as well. Thus, the clinical development of these molecules has been somewhat limited to date.

## Wnt/β-catenin as a biomarker for resistance to immunotherapy

Immunotherapy has become the first-line treatment strategy against advanced HCC (9). As we discussed above, in the IMbrave150 phase III clinical trial for advanced-stage HCC patients, the combination of the anti–PD-L1 antibody atezolizumab and the anti-VEGF antibody bevacizumab demonstrated an objective response rate of 36% (8). Unfortunately, ICIs have limited efficacy as monotherapy against HCC. For instance, the anti–PD-1 monoclonal nivolumab failed to improve HCC patient survival versus sorafenib in the phase III CheckMate 459 trial (9). One of the primary reasons for the failure of these clinical trials is that no biomarker-based patient selection has been implemented. Therefore, it is plausible to hypothesize that some patients harbor genetic events that confer resistance to ICIs. In this regard, aberrant activation of Wnt/β-catenin has emerged as an important pathway mediating ICI resistance (138, 139). Harding et al. reported that in HCC patients treated with ICIs, activation of the Wnt/β-catenin pathway correlates with lower disease control rate and lower progression-free and overall survival rates (140). Furthermore, studies using mouse HCC models confirmed that upregulated Wnt/β-catenin signaling in HCC promotes immune evasion and confers resistance to anti–PD-1 therapy (141). Mechanistically, it was found that activated β-catenin inhibits CCL5 expression, leading to impaired dendritic cell recruitment. Likewise, activated β-catenin in melanoma cells enhances ATF3 expression and subsequently represses CCL4 expression, leading to reduced recruitment of dendritic cells and consequently T cells into the tumor tissues (142). These findings suggest that *CTNNB1* mutational status could represent a novel biomarker for HCC patient exclusion for ICI treatment. Nevertheless, more studies are required to address the roles of the Wnt/β-catenin pathway in immunotherapy. For example, what is the Wnt/β-catenin mutation status in the IMbrave150 phase III clinical trial? Does the mutation status correspond to insensitivity to the combination immunotherapy or eventual progression over the treatment? Studies have suggested that NASH-related HCCs are particularly resistant to immunotherapies (143). Because the status of the Wnt/β-catenin pathway in NASH-related HCCs has not been well characterized, this question should be addressed using human HCC tissues and preclinical approaches.

## Challenges and future directions

Despite extensive studies on the Wnt/β-catenin cascade during hepatocarcinogenesis, our understanding of the molecular pathways deregulated by activated Wnt/β-catenin and how we can effectively target Wnt/β-catenin remains quite limited. Here, we discuss several key issues that need to be addressed to guide us for precision medicine.

### GOF CTNNB1 mutations and LOF AXIN1 mutations: same or different?

As we discussed above, both GOF *CTNNB1* mutations and loss-of-function (LOF) *AXIN1* mutations promote canonical Wnt pathway activation in HCC (59). Genetic studies have shown that these two mutations are mutually exclusive in human HCCs (Figure 1A), further supporting that they likely function via the major common pathway during hepatocarcinogenesis. Intriguingly, considerable differences have also been revealed based on recent genomic studies (Table 2). Specifically, HCCs with GOF *CTNNB1* mutations belong to the nonproliferation group, whereas HCCs with LOF *AXIN1* mutations are classified into the proliferation group (40). Additional molecular analysis revealed that *AXIN1*-mutant HCCs show relatively low canonical Wnt pathway activation levels but higher YAP/NOTCH induction, while *CTNNB1*-mutant HCCs show robust canonical Wnt pathway and mTOR signaling activation (144). These data suggest that GOF *CTNNB1* and LOF *AXIN1* might induce overlapping but also distinct downstream molecular events during hepatocarcinogenesis. It is tempting to hypothesize that LOF *AXIN1*-mutant HCCs, but not GOF *CTNNB1*-mutant tumors, depend on the YAP cascade for growth. If so, we need to understand how YAP becomes activated downstream of LOF *AXIN1*, and whether targeting YAP, such as using TNKS inhibitors, will lead to regression of HCC with LOF *AXIN1* mutations.

### What is the role of canonical Wnt/β-catenin signaling in HCCs in the absence of AXIN1 or CTNNB1 mutations?

Based on the published data and the recent genomic studies, such as TCGA analysis, it is clear that Wnt ligands and their receptors are frequently upregulated in human HCC samples. However, one can also clearly see that high expression of canonical Wnt target genes, including *GLUL* (encoding GS) and *TBX3*, tracks strongly with GOF *CTNNB1* mutations in human HCC samples (Figure 1A). Therefore, upregulation of Wnt ligands/receptors obviously does not induce strong activation of the canonical Wnt/β-catenin pathway. What is the functional role of the canonical Wnt/β-catenin cascade during HCC molecular pathogenesis in the absence of *AXIN1* or *CTNNB1* mutations? Most studies so far have relied on HCC cell lines (60, 113, 145). However, studies have suggested that Wnt ligands are likely to be produced by cells within the microenvironment. For example, in the normal liver, Wnts are secreted from sinusoid endothelial cells (146) or Kupffer cells during liver regeneration (147). The cellular sources of Wnt ligands in HCC remain to be defined. If they are secreted by the cells within the tumor microenvironment, it would be essential to investigate this canonical Wnt/β-catenin signaling in HCC when tumor cells are in their appropriate context, such as using murine HCC models. This question is critical to determine whether targeting of Wnt ligands, such as with PORCN inhibitors, may help to treat HCC without *AXIN1* or *CTNNB1* mutations.

### Is mTOR inhibition effective for the treatment of HCCs with GOF CTNNB1 mutations?

As we discussed above, activated β-catenin leads to mTORC1 activation, and mouse HCCs with GOF *Ctnnb1* mutations are sensitive to mTOR inhibition (90). On the other hand, monotherapy of everolimus, an mTOR inhibitor, has limited efficacy against advanced HCC (148). However, no biomarker-based patient selection was conducted in this clinical trial. This issue represents a major drawback of the trial, as the mTOR pathway is modulated by multiple cascades in cancer (149), including HCC (150); in addition, HCC is a highly heterogeneous disease. One can envision that the selection of patients with GOF *CTNNB1* mutations might be helpful to demonstrate the clinical efficacy of this drug. Furthermore, everolimus is a first-generation and partial mTORC1 inhibitor. The second-generation mTOR inhibitors, including mTORC1/mTORC2 inhibitors and mTOR/PI3K inhibitors, might have improved efficacy against HCCs with GOF *CTNNB1* mutations (151). Additional preclinical and clinical studies are required to address this critical issue.

### Can gene editing to reverse CTNNB1 mutation be useful for HCC treatment?

Recent progress with CRISPR/Cas9–based gene editing technology opens the door to genetic modification of tumor cells. GOF *CTNNB1* mutations, especially point mutations, are attractive targets for such a gene editing approach for cancer treatment. One can imagine delivering the proper guide RNA into HCC cells and reversing the mutant form of the *CTNNB1* allele into the wild-type sequence. However, small molecules directly targeting Wnt/β-catenin are frequently associated with significant gastrointestinal toxicity, as Wnt/β-catenin is necessary for intestinal stem cell renewal and proliferation. This toxicity substantially limits the clinical application of these small molecules. The gene editing approach has the advantage of not affecting the Wnt/β-catenin pathway in any other cells besides HCC cells that harbor the *CTNNB1* mutations. However, we do not know whether conversion into the wild-type *CTNNB1* sequence will lead to HCC regression, since wild-type β-catenin may be sufficient to support HCC progression. In addition, an efficient delivery method so that the guide RNAs can target all HCC cells should be developed.

### Wnt inhibitors: monotherapy or combination therapy?

As we discussed above, animal studies have demonstrated that the activation of Wnt/β-catenin alone is insufficient to promote HCC development. Instead, a second oncogenic signal is required for liver tumor formation (Table 1). Therefore, it is conceivable that targeting Wnt/β-catenin alone, either directly or indirectly (such as with mTOR inhibitors), is not sufficient to induce tumor regression. In contrast, combination therapies that target multiple signaling cascades might be required for efficient therapeutics. This point is highlighted by a recent study in murine HCC models coexpressing c-Met and ΔN90–β-catenin proto-oncogenes. In these mice, combined treatment with cabozantinib, which targets c-Met, and the dual mTOR inhibitor MLN0128, which targets activated β-catenin effectors, leads to tumor regression, whereas cabozantinib or MLN0128 monotherapy does not (152). As HCC is a heterogeneous disease, it would be critical to determine the specific pathways aberrantly activated in each HCC. Then one could design effective anti–Wnt/β-catenin–based combination therapies.

In summary, in the era of precision medicine, we can readily detect HCCs harboring activated Wnt/β-catenin signaling. These HCCs have peculiar molecular and pathological features and might be treated with effective and specific targeted therapies. However, our understanding of how the Wnt/β-catenin pathway contributes to HCC molecular pathogenesis remains incomplete. Therefore, additional molecular and biochemical studies are required to investigate this vital issue to identify novel targeted therapies against HCC with aberrant Wnt/β-catenin activation.

## Figures and Tables

**Table 2:**
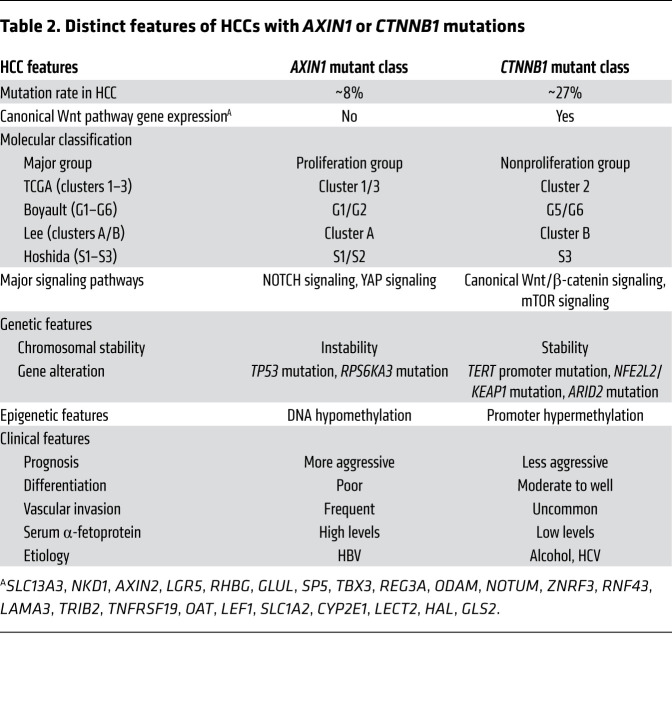
Distinct features of HCCs with *AXIN1* or *CTNNB1* mutations

**Table 1:**
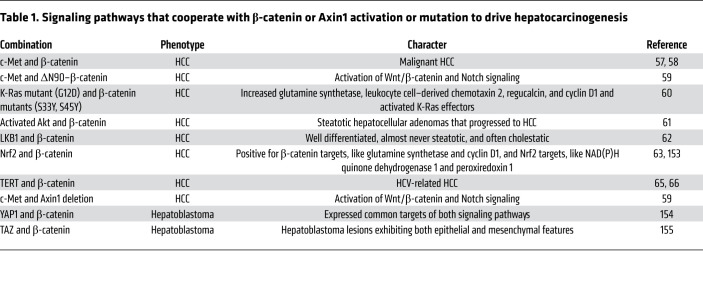
Signaling pathways that cooperate with β-catenin or Axin1 activation or mutation to drive hepatocarcinogenesis

## Version 1 02/15/2022

Electronic publication
